# First neonates with severe acute respiratory syndrome coronavirus 2 infection in Romania: Three case reports

**DOI:** 10.1097/MD.0000000000021284

**Published:** 2020-08-14

**Authors:** Mirabela Dima, Ileana Enatescu, Marius Craina, Izabella Petre, Emil Radu Iacob, Daniela Iacob

**Affiliations:** a Department of Neonatology; b Department of Obstetrics and Gynecology; c Department of Pediatric Surgery, “Victor Babes” University of Medicine and Pharmacy, Timisoara, Romania.

**Keywords:** diaper erythema, neonates, oral candidiasis, severe acute respiratory syndrome coronavirus 2, thrombocytopenia

## Abstract

**Rationale:**

The severe acute respiratory syndrome coronavirus 2 (SARS-CoV-2) pandemic, which quickly spread throughout the world, has been putting medical workers all over the world in difficulty because of the high number of cases combined with the lack of information about the disease. Although pediatric cases are rare, the group age under 12 months has been in general more susceptible to develop severe forms of the disease compared with the patients in the age interval of 1 to 18 years.

**Patient concerns:**

Three newborns have been tested positive for SARS-CoV-2 infection. One of them presented bilateral decreased air entry, while the other 2 had no respiratory symptomatology. All 3 developed diaper erythema and oral candidiasis.

**Diagnosis:**

For building up the report, newborns that were positive for coronavirus disease 2019 (COVID-19) infection were included in the case series. The chest X-ray of the symptomatic patient revealed a medium degree of hilar parenchymal infiltration and a slight infiltration of the visceral pleura.

**Interventions:**

The patients were admitted in our isolated neonatology ward. All of them received antifungal treatment for the oral candidiasis and topic cream for diaper erythema. The symptomatic patient also received prophylactic antibiotherapy, human immunoglobulins, aminophylline, and parenteral nutrition.

**Outcomes:**

All 3 neonates were discharged after 2 consecutive negative tests for SARS-CoV-2. Patients 1 and 2 fully recovered, whereas the condition of patient 3 improved.

**Lessons:**

Even if there are only a few reported cases of neonates infected with COVID-19 and most of them present mild manifestations, newborns need a more careful insight because of the nonspecific symptomatology.

## 1 Introduction

The novel coronavirus officially named severe acute respiratory syndrome coronavirus 2 (SARS-CoV-2) by the International Committee on Taxonomy of Virus generated a pandemic, which erupted in Hubei, Wuhan, China and quickly spread throughout the world,^[1,2]^ has been putting medical workers all over the world in difficulty because of the high number of cases combined with the lack of information about the disease.

As of May 2020, most of the existing literature approaches adult patients, especially those with comorbidities because this category of patients has been the most vulnerable to coronavirus disease 2019 (COVID-19).^[3]^

The pediatric group seems to be infrequently affected, furthermore aside the uncommonness of the cases, in most of them symptomatology is nonexistent or mild.^[4,5]^

Although pediatric cases are rare, the group age under 12 months has been in general more susceptible to develop severe forms of the disease compared with the patients in the age interval of 1 to 18 years.^[5]^ For this reason, in our opinion, neonates need a more careful look.

Despite the fact that neonates are usually asymptomatic or with mild symptomatology, extremely rare cases of Kawasaki-like syndrome^[6]^ or other severe affections have been reported.^[7]^

We will further present you 3 cases of neonates born in a clinic that in the period of their births was under suspicion of a SARS-CoV-2 outbreak throughout the ranks of the patients. Even if on discharge both mothers and newborns were tested negative, during the follow-up period, their SARS-CoV-2 polymerase chain reaction (PCR) tests turned out positive. Therefore, they were admitted in our isolated neonatology department.

This study was approved by the Ethics Committee of the Timiş County Emergency Clinical Hospital “Pius Brinzeu” and all parents signed a written informed consent form, both for treatment and for using their data in scientific papers.

## 2 Case reports

### 2.1 Patient 1

Full-term male newborn, delivered on 28th of March 2020 with birth weight of 3440 g, length 53 cm and head circumference 35 cm, APGAR score was 10 at 1 minute. The clinic where the patient was born discharged him and the mother after being tested negative for SARS-CoV-2.

On April 12, in consequence of the follow-up reverse transcription PCR (RT-PCR) test which turned out positive the infant was brought from home by ambulance and admitted in isolation, although he was not presenting any characteristic symptomatology. At this moment, no other family member except for the mother was tested positive or being symptomatic.

Initial examination showed good general condition, good tonus and reactivity, mild jaundice, and body temperature of 37.3°C (infrared electric body thermometer). There were no other pathologic findings, except for the left cryptorchidism and bilateral thallus valgus. His heart rate (HR) was 120 bpm, SaO_2_ (oxygen saturation) 97%, mean arterial pressure (MAP) = 71, and a respiratory rate (RR) of 44 per minute.

**Figure 1:**
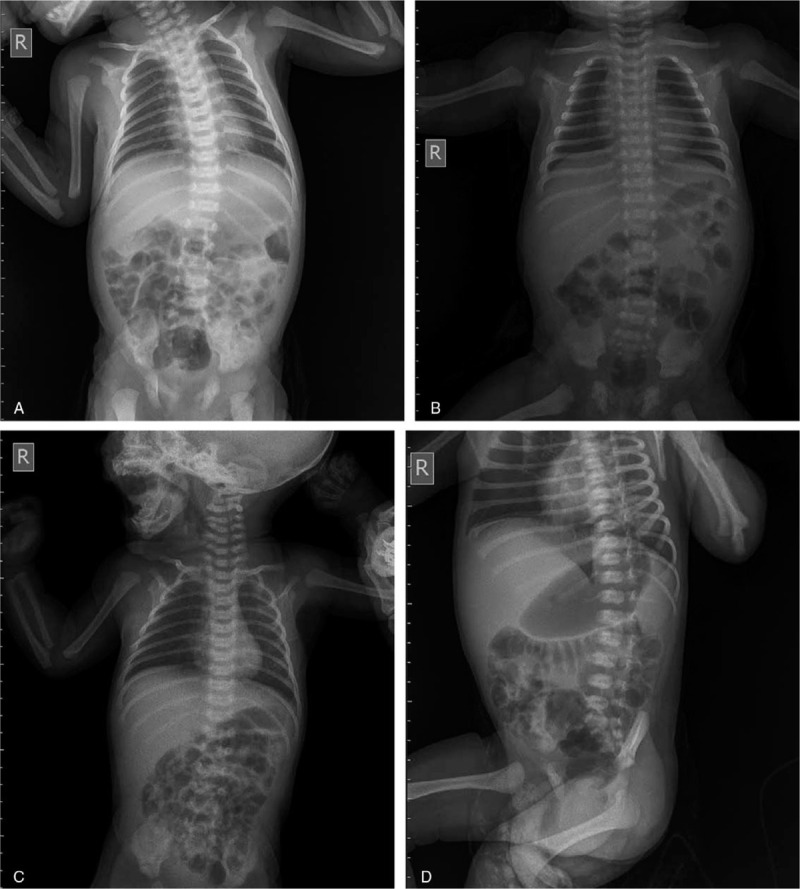
(A) Chest X-ray of patient 1 on admission, showing normal radiologic appearance of lungs. (B) Chest X-ray of patient 2 on admission, showing normal radiologic appearance of lungs. (C) Chest X-ray of patient 3 on admission – medium degree of hilar parenchymal infiltration and a slight infiltration of the visceral pleura. (D) Chest X-ray of patient 3 on discharge, normal radiologic appearance of lungs, with no pathologic signs.

None of the laboratory blood work revealed any relevant modification for our diagnosis. The initial chest X-ray performed on admission revealed normal radiologic appearance of lungs with no other pathologic findings (Fig. 1A).

During hospitalization, his general state continued to be good; however, he developed oral candidiasis, diaper erythema and epistaxis when the swab specimen was collected. His treatment included vitamin D, topical cream for erythema and Nystatin for the oral candidiasis.

Regarding SARS-CoV-2 PCR tests, on day 14 the result was inconclusive, then on day 15, he was negative. After 24 hours we collected another test according to our national protocol and the result was again inconclusive. During days 17 and 18 of hospitalization, we obtained 2 consecutive negative results, the patient being ready for discharge.

### 2.2 Patient 2

A full-term male born on April 2, 2020 with birth weight 4230 g, length 53 cm, and head circumference 38 cm, APGAR score 10 at 1 minute. The clinic where the patient was born discharged him and the mother after being tested negative for SARS-CoV-2.

He was presented 10 days after birth in the pediatric emergency department of another hospital for bilateral purulent eye discharge and fever. A RT-PCR test for SARS-CoV-2 was performed for both mother and infant and their results were positive. At this moment, no other family member was tested positive or being symptomatic.

On admission in our isolated neonatology ward, he had impaired general state and was unsettled. Heart and lung auscultation revealed no pathologic sounds. In addition to the clinical manifestations he presented in the emergency room we also noticed pale and marmorated skin and palpebral edema. His body temperature was 37.1°C, HR = 130 beats/min and SaO_2_ 97%, MAP = 59 and RR = 52.

None of the laboratory blood work exposed any relevant modification for our diagnosis and the initial chest X-ray revealed normal radiologic appearance of lungs with no other pathologic findings (Fig. 1B).

During hospitalization, he developed oral candidiasis, diaper erythema, and epistaxis when swab specimen was collected. In what concerns the treatment he received vitamins, eye drops, topical cream for erythema, and Nystatin for the oral candidiasis.

Regarding SARS-CoV-2 PCR tests, on day 14, the result was inconclusive, then on day 15, he was positive again. After 7 days, we collected another swab that showed a negative result followed by another negative test 24 hours later. The infant was discharged from the hospital along with the mother, who was also negative on 2 consecutive tests.

### 2.3 Patient 3

A female full-term neonate, born on March 31, with birth weight of 3060 g, length 49 cm, and head circumference 35 cm, APGAR score of 10 at 1 minute. The clinic where the patient was born discharged her and the mother on April 6, 2020 both being negative for SARS-CoV-2 (RT-PCR test).

On April 15, after 3 days of observing cough, lethargy, loss of appetite, jaundice, and constant fever, the mother presented in emergency room with the newborn, both being tested positive for SARS-CoV-2. At the same time, her father experienced fever, diarrhea, coughing, loss of smell, and taste. He was also tested positive for SARS-Cov-2.

The next day, the patient was admitted to our isolated neonatology ward. On initial examination, the newborn had impaired general condition with generalized hypotonia. She lost 200 g since birth, had lazy skin fold, generalized marmorated skin, and perioral and perinasal cyanosis. The neonate also presented diaper erythema and oral candidiasis.

Lung auscultation revealed bilateral decreased air entry, with no pathologic sounds and normal heart auscultation, HR of 140 bpm, SaO_2_ = 92%, MAP = 58, RR = 65, and body temperature 37.5°C. There were no other concerning clinical findings.

None of the laboratory blood work revealed any relevant modification for our diagnosis on admission day, but during hospitalization the patient developed anemia and transient thrombocytopenia. We had to change the cannula on a daily basis because of the ruptured blood vessels that made the needles unusable and resulted in multiple skin ecchymoses around the puncture sites.

The initial chest X-ray revealed a medium degree of hilar parenchymal infiltration and a slight infiltration of the visceral pleura (Fig. 1C).

During the first 4 days of hospitalization her general condition was unchanged, presenting fever (38.8°C) and coughing, despite her undergoing empirical antibiotic treatment with Ampicillin (100 mg/kgc/d) and Gentamicin (4 mg/kgc/d) and human immunoglobulins considering the severe thrombocytopenia. She was also given aminophylline (3 × 0.3 mL/d) to ease pulmonary effort, topic cream with Nystatin for the diaper erythema. For the treatment of the oral candidiasis and prevention of other fungal infection, she received Fluconazole iv (6 mg/kgc) and oral coating with Nystatin. Due to her loss of appetite, she received parenteral nutrition.

After the 4th day of treatment, her condition slowly improved. She started gaining weight, the diaper erythema and oral candidiasis were in obvious remission and the coughing and fever episodes ended.

**Figure 2:**
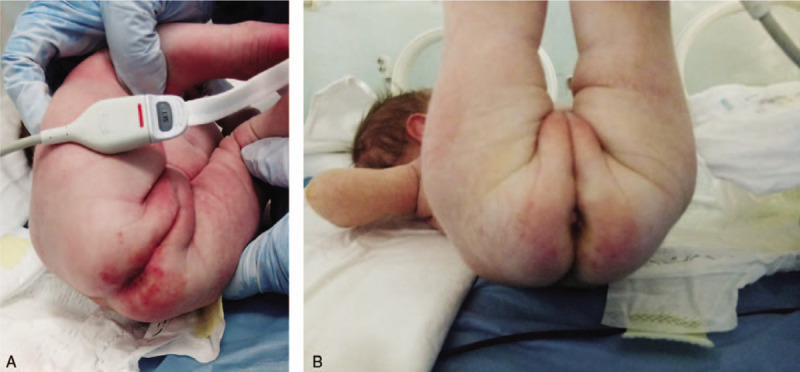
Patient 3. (A) Diaper erythema at the beginning of treatment. (B) Signs of remission of the diaper erythema.

On day 14 of hospitalization, lung and heart clinical examination was within normal parameters, her chest X-ray showed normal radiologic appearance of lungs with no pathologic signs (Fig. 1D) and the diaper erythema was in remission (Fig. 2A, B). On the days 15 and 16, she tested negative for SARS-CoV-2, but was kept under observation until her mother was also tested negative and were both discharged on May 6 (after 21 days of hospitalization) with the newborn being in healthy condition and weighting 3270 g.

In Table 1, the clinical, laboratory, and X-ray data for all 3 patients are summarized.

**Table 1:**
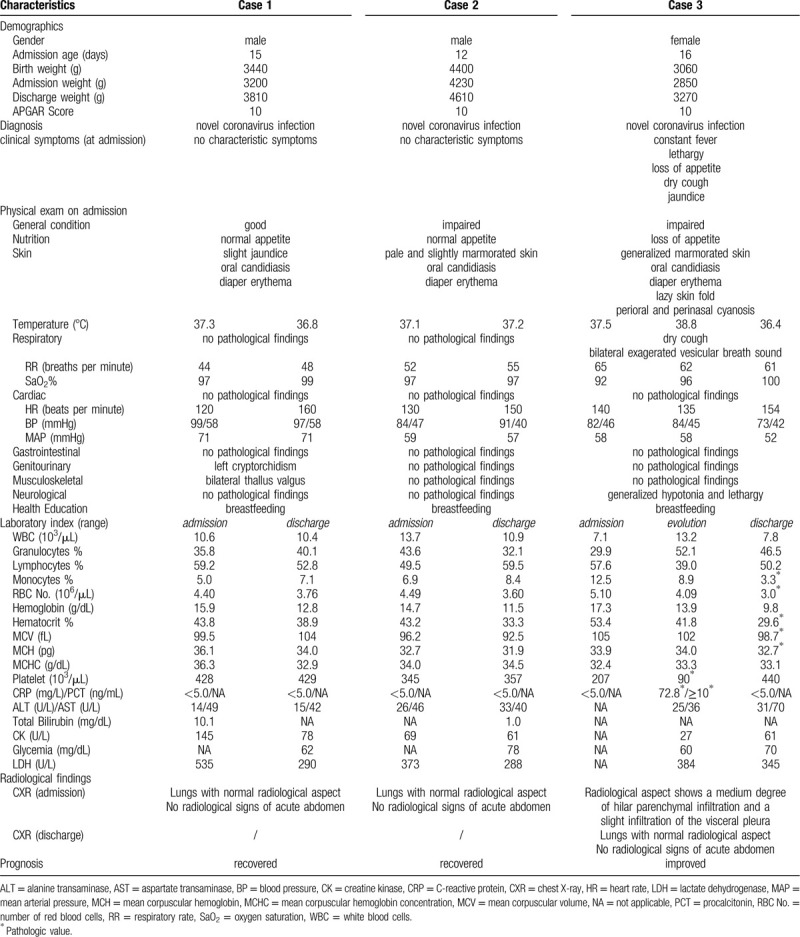
Characteristics of three neonates with SARS-CoV-2 infection.

We mention that no antiviral treatment was initiated for the newborns in case, since none of them experienced severe symptoms. Also, during their time at home, before being admitted on our neonatology clinic, all the mothers breastfed the newborns.

After discharging the patients, we asked feedback by phone from all 3 mothers and none of them reported any further manifestations regarding the infants. Their follow-up blood work was in normal parameters except for patient 3, who still presented anemia but with obvious amelioration, in consequence of the treatment prescribed at discharge.

## 3 Discussion

In the context of the ongoing SARS-CoV-2 pandemic, the attention of both clinicians and researchers has been mainly focused on the implications of the disease in adults and elders, especially those with associated pathology.^[3]^

The literature that approaches the pediatric group age is limited at the moment, in consequence of the fact that this group age accounts for only 1% to 5% of the cases worldwide.^[8]^

Because of the atypical neonatal infection symptoms and the lack of relevant reports, it is easy to ignore neonatal infections as the first neonatal infection was reported on February 12 even if the spread of the virus began 2 months earlier.^[9]^

In Romania, at the moment the official national registry for COVID-19 cases states that the group age 0 to 9 years accounts for approximately 2.5% of the cases.^[10]^

We believe it is important in the current epidemiologic context to mention that all 3 patients were discharged from the clinic where they were born with SARS-CoV-2 negative tests (RT-PCR), which were taken in conformity with our national protocol regarding COVID-19. The negative results of the tests exclude the possibility of vertical transmission in our cases, which concords with the existing literature that says this type of transmission is very unlikely to happen, but has not yet been proved to be impossible.^[11–13]^

In only 1 case, we found a family member positive for SARS-CoV-2 (the father of patient 3), who has been presenting diarrhea, cough, fever, and loss of smell and taste. This elucidates the epidemiologic problem regarding this patient, but the transmission way for the other 2 is still unclear. After being discharged from the clinic of birth, no restrictions related to breastfeeding were advised to the mother by the treating doctor so they naturally fed their children. The presence of SARS-CoV-2 in breastmilk has been proved by identifying different parts of his genome (ORF1b and N) with RT-PCR methods,^[14]^ but the possibility of a mother infecting her child through breastfeeding is yet to be proved, as milk components might damage the viral ribonucleic acid.^[15]^

Although all patients were discharged with negative tests, thanks to the surveillance protocol practiced in our country, they have been tested in a follow-up of the cases and we managed to identify patients 1 and 2 as being positive even if they were asymptomatic. According to the same protocol, we admitted all 3 patients in our neonatology department where they were completely isolated from their mothers during hospitalization, in incubators with controlled humidity and temperature, under permanent observation by the medical health workers.

During their time in the hospital, 2 of the patients had epistaxis when the nasal swab was taken. Patient 3 developed multiple ecchymoses on puncture sites and her blood cannula had to be changed on a regular basis as it became unusable due to the ruptured blood vessels. The same patient developed during hospitalization a transient thrombocytopenia which resolved before discharge.

The virus mechanism for entering the human cells uses the angiotensin converting enzyme 2 binding site.^[16]^ This type of receptor is highly expressed in alveolar cells, myocytes, and vascular endothelium which might suggest the vascular fragility.^[17]^

Thrombocytopenia and high level of D-dimer have been the most constant haemostatic abnormalities related to COVID-19.^[18,19]^ Regarding other coagulation tests, the existing literature is contradictory.^[20–22]^

All of our patients presented either on admission or during hospitalization diaper erythema and oral candidiasis. Even if both are regarded as nonspecific symptoms, their mention could be relevant as no other author has associated these 2 clinical entities neonatal COVID-19 infection.

Although it is known that antibiotics can cause candidiasis,^[23]^ only patient 3 received antibiotics but she presented the fungal infection prior to the treatment administration. Therefore, we cannot consider it a cause of developing oral candidiasis.

According to the literature, the incidence of candidiasis among preterm newborns range between 3% and 23%.^[24]^ In our neonatology ward, the incidence of thrush was 4% in preterm newborns and only 0.25% in on-term neonates. In a context in which all of our SARS-CoV-2 positive patients developed oral candidiasis, it raises a question for future studies on how COVID-19 disease could facilitate the infection with *Candida albicans*.

**Figure 3:**
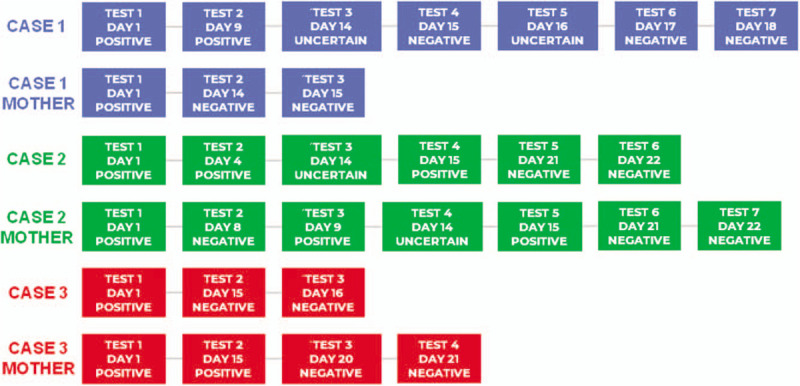
Flowchart showing the reverse transcription polymerase chain reaction test results of the newborns and their mothers.

One curious aspect about these 3 cases is the timeline of their RT-PCR tests (Fig. 3) where we can observe that the period in which the symptomatic patient was positive is shorter compared to the other 2. Furthermore, even if the mother of patient 3 was asymptomatic, her tests became negative 5 days later.

## 4 Conclusion

Because the existing literature on the subject is so scarce there are no well defined clinical patterns or implications. Nonspecific symptoms like diaper erythema and oral candidiasis were not yet seen in association with neonate COVID-19 infection.

Signs and symptoms that can suggest coagulation problems like thrombocytopenia, ecchymoses, and epistaxis have been too rarely reported in neonates to clearly define an interrelationship between the 2, even if in adult patients this correlation is delineated better. To better understand, the disease spectrum and possible clinical manifestations of COVID-19 in neonates, more comprehensive studies are needed.

## Acknowledgment

The authors thank the students Andrea Pasquini, Roxana Iacob, and Andrei Riza for their help in collecting data, designing table, and drawing pictures.

## Author contributions

**Conceptualization:** Mirabela Dima and Daniela Iacob.

**Resources:** Ileana Enatescu and Marius Craina.

**Supervision:** Daniela Iacob.

**Visualization:** Izabella Petre.

**Writing – original draft:** Mirabela Dima.

**Writing – review & editing:** Daniela Iacob and Emil Radu Iacob.
